# Genotype–phenotype associations of polymorphisms within the gene locus of NOD-like receptor pyrin domain containing 3 in Swiss inflammatory bowel disease patients

**DOI:** 10.1186/s12876-021-01880-9

**Published:** 2021-08-03

**Authors:** Priyatharsan Yoganathan, Jean-Benoit Rossel, Sebastian Bruno Ulrich Jordi, Yannick Franc, Luc Biedermann, Benjamin Misselwitz, Martin Hausmann, Gerhard Rogler, Michael Scharl, Isabelle Frey-Wagner, Swiss IBD cohort study group, Karim Abdelrahman, Gentiana Ademi, Patrick Aepli, Amman Thomas, Claudia Anderegg, Anca-Teodora Antonino, Eva Archanioti, Eviano Arrigoni, Diana Bakker de Jong, Bruno Balsiger, Polat Bastürk, Peter Bauerfeind, Andrea Becocci, Dominique Belli, José M. Bengoa, Luc Biedermann, Janek Binek, Mirjam Blattmann, Stephan Boehm, Tujana Boldanova, Jan Borovicka, Christian P. Braegger, Stephan Brand, Lukas Brügger, Simon Brunner, Patrick Bühr, Bernard Burnand, Sabine Burk, Emanuel Burri, Sophie Buyse, Dahlia-Thao Cao, Ove Carstens, Dominique H. Criblez, Sophie Cunningham, Fabrizia D’Angelo, Philippe de Saussure, Lukas Degen, Joakim Delarive, Christopher Doerig, Barbara Dora, Susan Drerup, Mara Egger, Ali El-Wafa, Matthias Engelmann, Jessica Ezri, Christian Felley, Markus Fliegner, Nicolas Fournier, Montserrat Fraga, Yannick Franc, Pascal Frei, Remus Frei, Michael Fried, Florian Froehlich, Raoul Ivano Furlano, Luca Garzoni, Martin Geyer, Laurent Girard, Marc Girardin, Delphine Golay, Ignaz Good, Ulrike Graf Bigler, Beat Gysi, Johannes Haarer, Marcel Halama, Janine Haldemann, Pius Heer, Benjamin Heimgartner, Beat Helbling, Peter Hengstler, Denise Herzog, Cyrill Hess, Roxane Hessler, Klaas Heyland, Thomas Hinterleitner, Claudia Hirschi, Petr Hruz, Pascal Juillerat, Carolina Khalid-de Bakker, Stephan Kayser, Céline Keller, Christina Knellwolf (-Grieger), Christoph Knoblauch, Henrik Köhler, Rebekka Koller, Claudia Krieger(-Grübel), Patrizia Künzler, Rachel Kusche, Frank Serge Lehmann, Andrew Macpherson, Michel H. Maillard, Michael Manz, Astrid Marot, Rémy Meier, Christa Meyenberger, Pamela Meyer, Pierre Michetti, Benjamin Misselwitz, Patrick Mosler, Christian Mottet, Christoph Müller, Beat Müllhaupt, Leilla Musso, Michaela Neagu, Cristina Nichita, Jan Niess, Andreas Nydegger, Nicole Obialo, Diana Ollo, Cassandra Oropesa, Ulrich Peter, Daniel Peternac, Laetitia Marie Petit, Valérie Pittet, Daniel Pohl, Marc Porzner, Claudia Preissler, Nadia Raschle, Ronald Rentsch, Alexandre Restellini, Sophie Restellini, Jean-Pierre Richterich, Frederic Ris, Branislav Risti, Marc Alain Ritz, Gerhard Rogler, Nina Röhrich, Jean-Benoît Rossel, Vanessa Rueger, Monica Rusticeanu, Markus Sagmeister, Gaby Saner, Bernhard Sauter, Mikael Sawatzki, Michael Scharl, Martin Schelling, Susanne Schibli, Hugo Schlauri, Dominique Schluckebier, Daniela Schmid, Sybille Schmid (-Uebelhart), Jean-François Schnegg, Alain Schoepfer, Vivianne Seematter, Frank Seibold, Mariam Seirafi, Gian-Marco Semadeni, Arne Senning, Christiane Sokollik, Joachim Sommer, Johannes Spalinger, Holger Spangenberger, Philippe Stadler, Peter Staub, Dominic Staudenmann, Volker Stenz, Michael Steuerwald, Alex Straumann, Bruno Strebel, Andreas Stulz, Michael Sulz, Aurora Tatu, Michela Tempia-Caliera, Joël Thorens, Kaspar Truninger, Radu Tutuian, Patrick Urfer, Stephan Vavricka, Francesco Viani, Jürg Vögtlin, Roland Von Känel, Dominique Vouillamoz, Rachel Vulliamy, Paul Wiesel, Reiner Wiest, Stefanie Wöhrle, Samuel Zamora, Silvan Zander, Tina Wylie, Jonas Zeitz, Dorothee Zimmermann

**Affiliations:** 1 Department of Gastroenterology and Hepatology, University Hospital and University of Zurich, Zurich, Switzerland; 2 Center for Primary Care and Public Health (Unisanté), University of Lausanne, Lausanne, Switzerland; 3 Department of Visceral Surgery and Medicine, Inselspital Bern and Berne University, Berne, Switzerland; 4 Zurich Center for Integrative Human Physiology, University of Zurich, Zurich, Switzerland; 5 Clinic for Gastroenterology and Hepatology, University Hospital Zurich, Rämistrasse 100, 8091 Zurich, Switzerland; 6 Present Address: Institute of Medical Microbiology, University of Zurich, Gloriastrasse 28/30, 8006 Zurich, Switzerland

**Keywords:** NLRP3 inflammasome, Inflammatory bowel disease, Single nucleotide polymorphisms, Clinical characteristics

## Abstract

**Background:**

Genetic variations within the regulatory region of the gene encoding NOD-like receptor pyrin domain containing 3 (*NLRP3*) have been associated with Crohn’s Disease (CD). NLRP3 is part of the NLRP3-inflammasome that mediates the maturation of IL-1β and IL-18. Carrying the major allele of the single nucleotide polymorphisms (SNPs) rs10733113, rs4353135 and rs55646866 is associated with an increased risk for CD. We here studied the impact of these polymorphisms on clinical characteristics in patients of the Swiss IBD Cohort Study (SIBDCS).

**Methods:**

We included 981 Crohn’s disease (CD) patients and 690 ulcerative colitis (UC) patients of the SIBDCS. We analyzed whether three CD-associated *NLRP3* polymorphisms have an impact on the clinical disease course in these patients.

**Results:**

In CD patients presence of the major allele (G) of rs10733113 was associated with less surgeries and lower maximal CDAI and a similar trend was observed for rs55646866 and rs4353135. Presence of the major allele of all three SNPs was negatively correlated to maximal CDAI. In UC patients homozygous genotype for the major allele (CC) for rs55646866 was associated with a higher age at diagnosis and a higher MTWAI index. Homozygous genotype for the major allele of all three polymorphisms was associated with a higher number of ambulatory visits and longer hospital stays.

**Conclusions:**

In CD patients presence of the major allele of all three polymorphisms was associated with markers of a less severe disease course, while in UC the homozygous genotype for all major alleles suggested a more severe disease activity.

**Supplementary Information:**

The online version contains supplementary material available at 10.1186/s12876-021-01880-9.

## Background

Genetic variations in a predicted regulatory region of the NOD-like receptor pyrin domain containing 3 (*NLRP3*) gene locus have been associated with an increased risk to develop Crohn’s disease (CD) [1]. CD risk has been linked to the major alleles of the *NLRP3* single nucleotide polymorphisms (SNPs) rs10733113, rs4353135 and rs55646866; however, 64–85% of the healthy European or Canadian population carry the disease-associated alleles [1]. For an overview of the major allele frequency in different populations and for details on SNP location see Additional file 2: Table S1 and Additional file 1: Figure S1.

The protein NLRP3 belongs to the family of nucleotide binding oligomerization domain (NOD) and leucine rich repeat (LRR) containing receptors (NLRs). It has an important role in the innate immune response, namely sensing of pathogen associated molecular patterns (PAMPs) and danger associated molecular patterns (DAMPs).

Upon activation, NLRP3 assembles with pro-caspase-1 and apoptosis-associated speck-like protein (ASC) to the NLRP3 inflammasome, resulting in autoproteolytic cleavage of pro-caspase-1 to active caspase-1 (CASP1). CASP1 is responsible for the conversion of pro-interleukin (IL)-1β and pro-IL-18 to the active cytokines [2]. Inflammatory bowel disease (IBD) patients tend to have higher levels of these cytokines in the serum [3]. Elevated secretion of IL-1β by lamina propria mononuclear cells isolated from inflamed IBD patients has been reported in several studies [4–6], and IL-1β levels in colonic perfusion fluid correlated with disease activity [7] or risk of relapse within the next year [8]. Yet, experimental colitis in mice deficient in NLRP3 could not prove an unequivocal pro-inflammatory role of NLRP3. Depending on the animal model, environment and hygienic conditions, lack of NLRP3 either ameliorated or aggravated experimental colitis [9–12].

So far, little is known about the functional consequences of human *NLRP3* associated genetic variants. The major alleles of rs4353135 and rs10733113 have been shown to lead to lower NLRP3 mRNA expression in human peripheral blood mononuclear cells (PBMCs) [1, 13]. The major allele (GG) of rs6672995 was associated with lower IL-1β secretion from stimulated PBMC [1], suggesting that the *NLRP3* polymorphisms that are associated with CD might lead to lower NLRP3-inflammasome activity. In line with the proposed pro-inflammatory role of the NLRP3 inflammasome during established IBD, polymorphisms that affect NLRP3 activity might also affect the clinical course of IBD patients, but such a relationship has not been addressed, so far.

We here studied for the first time whether the CD associated variants of the three SNPs (rs10733113, rs55646866, rs4353135) located in a regulatory region on chromosome 1q44 downstream of *NLRP3* affect the disease characteristics in IBD patients of the Swiss IBD cohort study (SIBDCS). In CD patients, the major allele of all three SNPs was negatively correlated to maximal CDAI, suggesting a less severe course of disease. In contrast, in UC patients, presence of the major allele of rs55646866 was associated with a higher MTWAI while homozygous genotype for the major allele of all three polymorphisms was associated with a higher number of ambulatory visits. Our findings might lead to a better understanding of disease pathogenesis depending on *NLRP3* genotype and identify patients that might benefit from a NLRP3 targeted therapy.

## Methods

### Patient data

Data were obtained from the nationwide Swiss Inflammatory Bowel Disease Cohort Study (SIBDCS). The SIBDCS is a prospective multicenter observational population-based study into which patients with IBD from all regions of Switzerland have been included since 2006. The goals and the methodology of the cohort have been described in detail previously [14, 15].

### Study design

We included 1671 IBD patients (981 CD and 690 UC) that were enrolled in the SIBDCS at time of data acquisition and had been genotyped for the CD-associated single nucleotide polymorphisms (SNP) rs4353134, rs55646866 and rs10733113 within a regulatory region on chromosome 1q44 downstream of the *NLRP3* gene. Genotyping was performed as part of an analysis of the whole SIBDCS for selected SNPs that are currently known to be associated with IBD [16]. SNP Genotyping of SIBDCS samples was performed by MALDI-TOFF based analysis [17].

The aim of this study was to analyze whether the CD-associated risk variants for *NLRP3* rs4353134, rs55646866 and rs10733113 are associated with clinical parameters defining the severity/course of disease. For rs4353134, the T-allele is the major allele and the G-allele the minor allele. The major and minor alleles for rs55646866 are C/T and for rs10733113 G/A, respectively.

For analysis of clinical characteristics, we included gender, diagnosis, age at diagnosis, disease duration, complications, Crohn’s disease activity index (CDAI) or modified Truelove and Witts activity index (MTWAI), reported flare (including possible cause of flare, flare management and hospitalization), fistula, abscess or anal fissure, stenosis, presence of extraintestinal manifestations, intestinal surgery and medication.

### Statistical analysis

Clinical data were obtained from the data center of the SIBDCS at the University of Lausanne. These data and additional data obtained from a review of the patients' files were entered into a database (Access 2000; Microsoft Switzerland Ltd Liab. Co., Wallisellen, Switzerland). Stata 14 software (StataCorp, 2015, College Station, TX was used for the statistical analysis.

We analyzed for associations between clinical characteristics and genotype for rs4353135, rs55646866 and rs10733113 with Fisher’s exact test for discrete variables and with the Kruskal–Wallis test for continuous variables. To analyze for a cumulative risk of the three variants, we calculated a score based on the odds ratio for the risk allele as described by Villani et al. [1] for all patients (Score = x * log(OR rs4353135 T-allele) + y * log(OR rs55646866 C-allele) + z * log (OR rs10733113 G-allele); x, y, z = number of major allele (0, 1 or 2)) resulting in a score between 0 and 2.58 for each patient. We performed linear regression analyses with this score as predictor and clinical parameters as response. Log(maxCDAI) was used for the analysis to reduce the impact of very high values.

For calculation of linkage disequilibrium (LD) we used the square of the correlation coefficient (r^2^) between SNP pairs (R, genetics package, with the function LD()) https://doi.org/10.1086/381000 [1].

## Results

In this study, we investigated a total of 1671 IBD patients of the SIBDCS. 981 (58.7%) patients were classified as CD and 690 (41.3%) as UC patients.

### Distribution of *NLRP3* SNP genotypes in CD and UC patients

The distribution of the alleles for rs4353135, rs55646866 and rs10733113713 and the joint distribution of alleles in CD and UC patients are presented in Tables 1 and 2. There were no significant differences in distribution of the genotypes between CD and UC patients. The major allele frequency in SIBDCS patients was 0.68 (T) for rs4353135, 0.89 (C) for rs55646866, and 0.74 (G) for rs10733113713, corresponding well with the major allele frequency in the European population. For an overview of the major allele frequencies in different populations see Additional file 2: Table S1. 422 (43.0%, CD) and 285 (41.3%, UC) patients are homozygous carriers for the major allele of all three SNPs. As CD and UC are distinct forms of IBD with diverging disease pathogenesis, we performed individual analyses for associations between *NLRP3* SNPs and disease characteristics in CD and UC patients.

**Table 1:** Cross table of joint SNP distribution in CD patients

	rs10733113			Total
	GG	AG	AA	
rs4353135: TT	423 (90.8%)	1 (0.2%)	42 (9.0%)	466 (100%)
GT	252 (61.5%)	5 (1.2%)	153 (37.3%)	410 (100%)
GG	38 (36.2%)	1 (0.9%)	66 (62.9%)	105 (100%)
Total	713 (72.7%)	7 (0.7%)	261 (26.6%)	981 (100%)
rs55646866: CC	710 (90.4%)	3 (0.4%)	72 (9.2%)	785 (100%)
CT	3 (1.7%)	4 (2.3%)	166 (96.0%)	173 (100%)
TT	0 (0%)	0 (0%)	23 (100%)	23 (100%)
Total	713 (72.7%)	7 (0.7%)	261 (26.6%)	981 (100%)

	rs4353135			Total
	TT	GT	GG	
rs55646866: CC	465 (59.2%)	282 (35.9%)	38 (4.8%)	785 (100%)
CT	1 (0.6%)	128 (74.0%)	44 (25.4%)	173 (100%)
TT	0 (0%)	0 (0%)	23 (100%)	23 (100%)
Total	466 (47.5%)	410 (41.8%)	105 (10.7%)	981 (100%)

**Table 2:** Cross table of joint SNP distribution in UC patients

	rs10733113			Total
	GG	AG	AA	
rs4353135: TT	286 (90.2%)	3 (1.0%)	28 (8.8%)	317 (100%)
GT	196 (65.5%)	11 (3.7%)	92 (30.8%)	299 (100%)
GG	34 (45.9%)	0 (0%)	40 (54.1%)	74 (100%)
Total	516 (74.8%)	14 (2.0%)	160 (23.2%)	690 (100%)
rs55646866: CC	514 (90.7%)	4 (0.7%)	49 (8.6%)	567 (100%)
CT	2 (1.8%)	10 (9.0%)	99 (89.2%)	111 (100%)
TT	0 (0%)	0 (0%)	12 (100%)	12 (100%)
Total	516 (74.8%)	14 (2.0%)	160 (23.2%)	690 (100%)

	rs4353135			Total
	TT	GT	GG	
rs55646866: CC	316 (55.7%)	216 (38.1%)	35 (6.2%)	567 (100%)
CT	1 (0.9%)	82 (73.9%)	28 (25.2%)	111 (100%)
TT	0 (0%)	1 (8.3%)	11 (91.7%)	12 (100%)
Total	217 (45.9%)	299 (43.3%)	74 (10.7%)	690 (100%)

### Presence of the major allele of all three SNPs is associated with lower maximal CDAI in CD patients

**Fig. 1:**
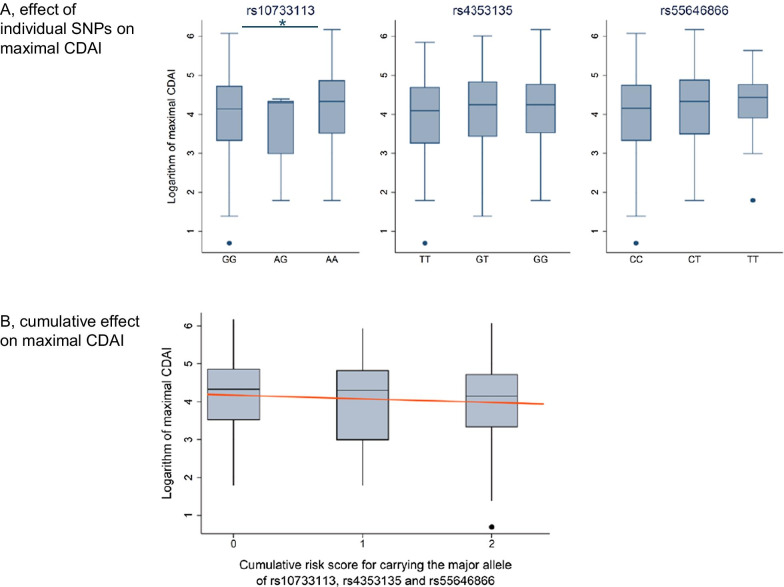
Effect of the major alleles of rs10733113, rs4353135 and rs55646866 on clinical parameters in CD patients. The graphs show median Log (max. CDAI) values for maximal CDAI within the 25% and 75% percentile (box borders) and outliers (dots) in CD patients **A** homozygous for the major allele, heterozygous or homozygous for the minor allele for rs10733113, rs4353135 and rs55646866, **B** depending on the cumulative genetic risk score for carrying the major allele for rs1073313, rs4353135 and rs55646866. In a linear regression model, a significant negative correlation between the cumulative genetic score and log (max. CDAI) can be observed (Coefficient − 0.097; 95% CI − 0.182 to − 0.011; *p* = 0.026) **C** CD duration in months within the 25% and 75% percentile (box borders) and outliers (dots) and **D** percentage of patients with stenosis that required an operation for treatment of stenosis in CD patients homozygous for the major allele, heterozygous or homozygous for the minor allele for rs10733113, rs4353135 and rs55646866

Presence of the major allele (G) of rs10733113 was associated with a significantly lower maximal CDAI value throughout follow-up (median CDAI = 63 in GG or AG vs. 76 in AA patients, *p* = 0.011, and we observed similar trends, yet not significant, for lower CDAI in carriers of the major alleles of rs4353135 and rs55646866 (Fig. 1A). The investigated SNPs have been reported to be linked due to close proximity of their loci [1] (they all lie within a 5321 bp region, for details see Additional file 1: Figure S1). In our cohort, however, the square of the correlation coefficient r^2^ (Additional file 2: Table S2) was below 0.2, suggesting no relevant linkage of the studied SNPs. To analyze whether there is an additive effect on the association between *NLRP3* SNPs and clinical parameters, we calculated a score for the cumulative genetic risk for carrying the major allele of all 3 polymorphisms. To calculate this score we added the log of the odds ratio (as identified by Villani et al. [1]) for the CD associated allele of the given SNP (log (1.78) = 0.58 for rs10733113, log (1.21) = 0.19 for rs43353135, log (1.69) = 0.52 for rs55646866) resulting in a score between 0 and 2.58 for each patient. In a linear regression model with this genetic score as predictor and log (max. CDAI) as response we observed a significant negative association (Coefficient − 0.097; 95% CI − 0.182 to − 0.011; *p* = 0.026), indicating that carrying the CD-associated alleles for the 3 investigated *NLRP3* SNPs is associated with a less severe course of disease as evaluated by disease symptoms (Fig. 1B), and that there might be an additive effect of the three SNPs.

### Carrying the major allele for rs55646844 is associated with longer disease duration in CD patients

Duration of disease, the time from diagnosis until last patient visit (i.e., the most recent patient visit, either at the regular yearly visit for the SIBDCS or the latest disease associated visit) 

was significantly longer in patients carrying the major allele for rs55646866 (12.3 years for CC or CT vs. 6.1 years for TT, *p* = 0.013). For rs4353135 a similar trend, yet not significant, could be observed (12.4 years for GT or TT vs. 9.9 years for GG, *p* = 0.090) while there was no effect for rs10733113. Yet, age at diagnosis was not affected by the genotype for rs55646844 (Fig. 1C).

### The major allele (GG) for rs10733113 is associated with fewer operations due to stenosis in CD patients

While the overall incidence of stenosis did not differ between genotypes, homozygous carriers of the major allele (GG) for rs10733113 had significantly less operations for treatment of stenosis (153 (49.4%) in GG, 0 (0%) in AG, 68 (58.1%) in AA, *p* = 0.045). The genotype of the SNPs rs4353235 and rs55646866 was not associated to the frequency of operations needed for treatment of stenosis (Fig. 1D).

### *NLRP3* SNPs are not associated with further clinical disease characteristics in CD patients.

The major alleles of rs10733113, rs4353235 and rs55646866 were associated with a lower maximal CDAI value, suggesting a less severe course of disease in carriers of the major alleles. We analyzed whether individual clinical characteristics e.g. flare frequency, extraintestinal manifestations or medication are associated with carrying the major allele of the investigated polymorphisms. No further associations between genotype for rs10733113, rs4353235, rs55646866 and disease characteristics were observed in CD patients. Tables 3, 4 and 5 show the demographic distribution and clinical characteristics investigated for the different genotypes of rs10733113, rs4353235 and rs55646866.

**Table 3:** Demographic and clinical characteristics for the different genotypes of rs10733113 in CD patients

	rs10733113 GG	rs10733113 AG	rs10733113 AA	*p* value (Fisher or Kruskal–Wallis)
*Gender*				
Male (n = 488)	363 (50.9%)	4 (57.1%)	121 (46.4%)	0.390
Female (n = 493)	350 (49.1%)	3 (42.9%)	140 (53.6%)	
*Age at diagnosis [years]*				
Median, q25–q75,	24.7, 18.1–34.8,	21.3, 15.3–32.2,	23.4, 17.6–32.1,	0.164
Min–max	1.1–81.4	9.0–40.1	0.8–81.0	
*Disease duration [years]*				
Median, q25–q75,	12.1, 7.0–20.7,	7.5, 5.4–16.9,	12.7, 6.8–23.5,	0.462
Min–max	0.1–56.6	3.4–20.5	0.3–52.4	
Complications*				
No (n = 345)	259 (36.3%)	4 (57.1%)	82 (31.4%)	0.185
Yes (n = 636)	454 (63.7%)	3 (42.9%)	179 (68.6%)	
Fistula, abscess or anal fissure*				
No (n = 518)	376 (52.7%)	4 (57.1%)	138 (52.9%)	1.000
Yes (n = 463)	337 (47.3%)	3 (42.9%)	123 (47.1%)	
*Focus on the “yes”:*				
Perianal fistula	180 (53.4%)	3 (100%)	60 (48.8%)	0.214
Other fistula	129 (38.3%)	1 (33.3%)	53 (43.1%)	0.686
Fissure	102 (30.3%)	1 (33.3%)	37 (30.1%)	1.000
Abscess	184 (54.6%)	1 (33.3%)	68 (55.3%)	0.779
Surgery for fistula	178 (52.8%)	1 (33.3%)	67 (54.5%)	0.753
Stenosis*				
No (n = 551)	403 (56.5%)	4 (57.1%)	144 (55.2%)	0.916
Yes (n = 430)	310 (43.5%)	3 (42.9%)	117 (44.8%)	
*Focus on the “yes”:*				
Operation	153 (49.4%)	0 (0%)	68 (58.1%)	0.045
Dilation	117 (37.7%)	2 (66.7%)	50 (42.7%)	0.359
No intervention	170 (54.8%)	1 (33.3%)	65 (55.6%)	0.772
CDAI**—maximal value throughout follow-up				
Median, q25**–**q75,	63, 28–112,	73, 20–76,	76, 33–129,	0.024
Min**–**max	2–435	6–81	0–479	
Reported flare*				
No (n = 477)	355 (49.8%)	4 (57.1%)	118 (45.2%)	0.369
Yes (n = 504)	358 (50.2%)	3 (42.9%)	143 (54.8%)	
*Flare possibly or highly related to (focus on yes):*				
NSAIDs	9 (2.5%)	0 (0%)	2 (1.4%)	0.753
Antibiotics	8 (2.2%)	0 (0%)	1 (0.7%)	0.485
GI tract infection	54 (15.1%)	0 (0%)	30 (21.0%)	0.203
Other infection	23 (6.4%)	0 (0%)	14 (9.8%)	0.357
Treatment decr./disc	89 (24.9%)	1 (33.3%)	44 (30.8%)	0.338
Other medication	4 (1.1%)	0 (0%)	3 (2.1%)	0.437
*Flare management (focus on the yes):*				
Hospitalization	61 (17.0%)	1 (33.3%)	24 (16.8%)	0.598
Ambulatory	88 (24.6%)	0 (0%)	41 (28.7%)	0.468
Surgery	37 (10.3%)	0 (0%)	13 (9.1%)	0.813
Drug therapy	308 (86.0%)	3 (100%)	127 (88.8%)	0.650
*Focus on the hospitalizations:*				
Total days of hosp.	7, 2–17,	0, 0–0,	4.5, 1–13.5,	0.386
Median, q25**–**q75,	0–55	0–0	0–86	
Min**–**max

**Table 4:** Demographic and clinical characteristics for the different genotypes of rs4353135 in CD patients

	rs4353135 TT	rs4353135 GT	rs4353135 GG	*p* value (Fisher or Kruskal–Wallis)
*Gender*				
Male (n = 488) Female (n = 493)	234 (50.2%) 232 (49.8%)	207 (50.5%) 203 (49.5%)	47 (44.8%) 58 (55.2%)	0.557
*Age at diagnosis [years]*				
Median, q25**–**q75, Min**–**max	24.4, 18.2–34.7, 1.1–81.0	23.9, 17.9–32.5, 0.8–81.4	25.2, 17.4–35.1, 1.6–73.7	0.465
*Disease duration [years]*				
Median, q25**–**q75, Min**–**max	12.4, 7.4–21.0, 0.1–52.0	12.2, 7.2–21.2, 0.2–56.6	9.9, 5.7–19.4, 1.2–44.0	0.239
Complications*				
No (n = 345) Yes (n = 636)	159 (34.1%) 307 (65.9%)	147 (35.9%) 263 (64.1%)	39 (37.1%) 66 (62.9%)	0.765
Fistula, abscess or anal fissure*				
No (n = 518) Yes (n = 463)	247 (53.0%) 219 (47.0%)	221 (53.9%) 189 (46.1%)	50 (47.6%) 55 (52.4%)	0.513
*Focus on the “yes”:*				
Perianal fistula Other fistula Fissure Abscess	117 (53.4%) 84 (36.4%) 66 (30.1%) 120 (54.8%)	93 (49.2%) 84 (44.4%) 52 (27.5%) 107 (56.6%)	33 (60.0%) 15 (27.3%) 22 (40.0%) 26 (47.3%)	0.356 0.066 0.207 0.474
*Surgery for fistula*	114 (52.1%)	102 (54.0%)	30 (54.5%)	0.913
Stenosis*				
No (n = 551) Yes (n = 430**)**	256 (54.9%) 210 (45.1%)	239 (58.3%) 171 (41.7%)	56 (53.3%) 49 (46.7%)	0.501
*Focus on the “yes”:*				
Operation Dilation No intervention	110 (52.4%) 81 (38.6%) 110 (52.4%)	83 (48.5%) 72 (42.1%) 98 (57.3%)	28 (57.1%) 16 (32.7%) 28 (57.1%)	0.541 0.469 0.595
*CDAI—maximal value throughout follow-up*				
Median, q25**–**q75, Min**–**max	60, 26–109, 0–345	70, 31–126, 4–405	70, 34–118, 6–479	0.071
Reported flare*				
No (n = 477) Yes (n = 504)	240 (51.5%) 226 (48.5%)	185 (45.1%) 225 (54.9%)	52 (49.5%) 53 (50.5%)	0.164
*Flare possibly or highly related to (focus on yes):*				
NSAIDs Antibiotics GI tract infection Other infection Treatment decr. / disc Other medication	5 (2.2%) 5 (2.2%) 35 (15.5%) 15 (5.8%) 50 (22.1%) 1 (0.4%)	4 (1.8%) 3 (1.3%) 42 (18.7%) 21 (9.3%) 70 (31.1%) 5 (2.2%)	2 (3.8%) 1 (1.9%) 7 (13.2%) 3 (5.7%) 14 (26.4%) 1 (1.9%)	0.613 0.710 0.545 0.347 0.097 0.180
*Flare management (focus on the yes):*				
Hospitalization Ambulatory Surgery Drug therapy	35 (15.5%) 59 (26.1%) 18 (8.0%) 198 (87.6%)	42 (18.7%) 59 (26.2%) 26 (11.6%) 195 (86.7%)	9 (17.0%) 11 (20.8%) 6 (11.3%) 45 (84.9%)	0.652 0.753 0.398 0.828
*Focus on the hospitalizations:*				
Total days of hosp. Median, q25**–**q75, Min**–**max	7, 0–12, 0–47	5.5, 2–17, 0–86	12, 3–20, 0–50	0.646

**Table 5:** Demographic and clinical characteristics for the different genotypes of rs55646866 in CD patients

	rs55646866 CC	rs55646866 CT	rs55646866 TT	*p* value (Fisher or Kruskal–Wallis)
*Gender*				
Male (n = 488) Female (n = 493)	394 (50.2%) 391 (49.8%)	81 (46.8%) 92 (53.2%)	13 (56.5%) 10 (43.5%)	0.605
*Age at diagnosis [years]*				
Median, q25**–**q75, Min**–**max	24.3, 17.9–34.4, 1.1–81.4	24.4, 18.5–33.0, 0.8–65.9	25.0, 18.7–31.5, 11.5–73.7	0.875
*Disease duration [years]*				
Median, q25**–**q75, Min**–**max	12.2, 7.0–20.9, 0.1–56.6	12.8, 7.6–23.5, 1.2–44.7	6.1, 4.5–13.1, 1.5–33.8	**0.030**
Complications*				
No (n = 345) Yes (n = 636)	281 (35.8%) 504 (64.2%)	56 (32.4%) 117 (67.6%)	8 (34.8%) 15 (65.2%)	0.727
Fistula, abscess or anal fissure*				
No (n = 518) Yes (n = 463)	411 (52.4%) 374 (47.6%)	94 (54.3%) 79 (45.7%)	13 (56.5%) 10 (43.5%)	0.836
*Focus on the “yes”:*				
Perianal fistula Other fistula Fissure Abscess	199 (53.2%) 144 (38.5%) 113 (30.2%) 203 (54.3%)	38 (48.1%) 36 (45.6%) 24 (30.4%) 44 (55.7%)	6 (60.0%) 3 (30.0%) 3 (30.0%) 6 (60.0%)	0.641 0.439 1.000 0.930
Surgery	195 (52.1%)	46 (58.2%)	5 (50.0%)	0.626
Stenosis*				
No (n = 551) Yes (n = 430)	446 (56.8%) 339 (43.2%)	95 (54.9%) 78 (45.1%)	10 (43.5%) 13 (56.5%)	0.413
*Focus on the “yes”:*				
Operation Dilation No intervention	169 (49.9%) 133 (39.2%) 189 (55.8%)	44 (56.4%) 33 (42.3%) 40 (51.3%)	8 (61.5%) 3 (23.1%) 7 (53.8%)	0.460 0.434 0.766
*CDAI—maximal value throughout follow-up*				
Median, q25**–**q75, Min**–**max	64, 28–114, 0–435	76, 33–131, 6–479	64, 50–117, 6–280	0.108
Reported flare*				
No (n = 477) Yes (n = 504)	387 (49.3%) 398 (50.7%)	82 (47.4%) 91 (52.6%)	8 (34.8%) 15 (65.2%)	0.365
*Flare possibly or highly related to (focus on yes):*				
NSAIDs Antibiotics GI tract infection Other infection Treatment decr./disc Other medication	8 (2.0%) 8 (2.0%) 63 (15.8%) 25 (6.3%) 100 (25.1%) 4 (1.0%)	2 (2.2%) 0 (0%) 20 (22.0%) 11 (12.1%) 29 (31.9%) 3 (3.3%)	1 (6.7%) 1 (6.7%) 1 (6.7%) 1 (6.7%) 5 (33.3%) 0 (0%)	0.450 0.154 0.255 0.143 0.319 0.292
*Flare management (focus on the yes):*				
Hospitalization Ambulatory Surgery Drug therapy	68 (17.1%) 96 (24.1%) 39 (9.8%) 344 (86.4%)	16 (17.6%) 31 (34.1%) 11 (12.1%) 81 (89.0%)	2 (13.3%) 2 (13.3%) 0 (0%) 13 (86.7%)	0.967 0.090 0.443 0.776
*Focus on the hospitalizations:*				
Total days of hosp. Median, q25**–**q75, Min**–**max	7.5, 2–16, 0–55	3.5, 0–17.5, 0–86	4, 3–5, 3–5	0.553

Bold indicates a significant *p*-value (*p* < 0.05)

### Carrying the major alleles for rs10733113, rs4353235 and rs55646866 is associated with a higher number of ambulatory hospitalizations in UC patients

**Fig. 2:**
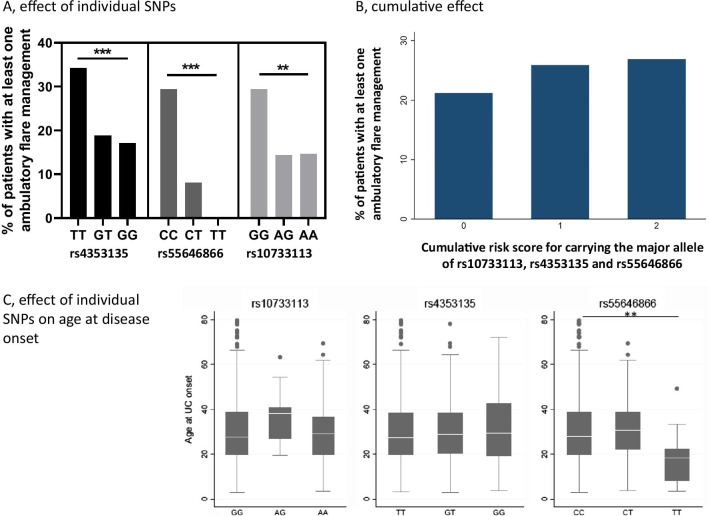
Individual and cumulative effect of the major alleles of rs10733113, rs4353135 and rs55646866 on ambulatory flare management in UC patients. The graphs show the percentage of UC patients with at least one ambulatory visit for flare management **A** Homozygous for the major allele, heterozygous or homozygous for the minor allele for rs10733113, rs4353135 and rs55646866. **B** Depending on the cumulative genetic risk score for carrying the major allele for rs10733113, rs4353135 and rs55646866. In a linear regression model a significant association between the cumulative genetic score and the percentage of patients needing ambulatory flare management could be observed (Odds ratio 2.081; 95% CI − 1.393 to 3.11; *p* < 0.001). **C** Age at disease onset is shown within the 25% and 75% percentile (box borders) and outliers (dots) in UC patients.

Additional ambulatory visits are strong indicators for occurrence of a moderate to severe flare in UC patients. Homozygous genotype for the major allele of all three polymorphisms was associated with a higher number of ambulatory visits for flare management in UC patients (Fig. 2A). Despite their close proximity and earlier reports of linkage disequilibrium, the investigated polymorphisms showed low linkage disequilibrium in the SIBDCS (Additional file 2: Table S2) and we observed a significant association between the number of risk-associated alleles and the number of visits. We applied the same genetic score as calculated for the cumulative effect of all three SNPs on CDAI in CD patients for analysis of ambulatory flare management in UC patients. Based on a logistic regression, we find that this score is significantly associated with the occurrence of at least one ambulatory flare measure (OR: 2.081 (95% CI 1.393–3.111; *p* < 0.001)) (Fig. 2B).

### Homozygous genotype for the major allele (CC) of rs55646866 is associated with a higher age at disease onset and a higher MTWAI index

The MTWAI index characterizes the severity of disease as defined by symptoms in UC patients. Homozygous genotype (CC) for rs55646866 was associated with a higher MTWAI index in UC patients (mean MTWAI: 5 (CC), 5 (CT), 1.5 (TT), *p* = 0.009). We further observed that homozygous genotype for the major allele of rs55646866 was associated with a higher age at diagnosis (mean age at diagnosis: 28 (CC), 30 (CT), 18.3 (TT), *p* = 0.004) (Fig. 2C).

### *NLRP3* SNPs are not associated with further clinical disease characteristics in UC patients.

The major alleles of rs10733113, rs4353235 and rs55646866 were associated with a higher number of ambulatory hospitalizations for flare management, suggesting a more severe course of disease in carriers of the major alleles. We therefore investigated whether individual clinical characteristics, e.g. flare frequency, extraintestinal manifestations or medication, that are usually affected during a more severe disease course, are associated with carrying the major allele for rs10733113, rs4353235 and rs55646866. No further associations between homozygous genotype for the major allele of rs10733113, rs4353235, rs55646866 and disease characteristics were observed in UC patients. Tables 6, 7 and 8 show the demographic distribution and clinical characteristics investigated for the different genotypes of rs10733113, rs4353235 and rs55646866.

**Table 6:** Demographic and clinical characteristics for the different genotypes of rs10733113 in UC patients

	rs10733113 GG	rs10733113 AG	rs10733113 AA	*p* value (Fisher or Kruskal–Wallis)
*Gender*				
Male (n = 369) Female (n = 321)	276 (53.5%) 240 (46.5%)	10 (71.4%) 4 (28.6%)	83 (51.9%) 77 (48.1%)	0.392
*Age at diagnosis [years]*				
Median, q25**–**q75, Min**–**max	27.7, 19.8–38.7, 2.9–79.6	38.0, 27.1–40.8, 19.5–63.2	29.2, 19.7–36.7, 3.4–69.4	0.099
*Disease duration [years]*				
Median, q25**–**q75, Min**–**max	11.1, 6.7–18.4, 0.2–49.5	10.7, 7.8–17.8, 2.9–26.3	11.7, 7.3–18.8, 0.5–40.1	0.619
Complications*				
No (n = 286) Yes (n = 404)	225 (43.6%) 291 (56.4%)	6 (42.9%) 8 (57.1%)	55 (34.4%) 105 (65.6%)	0.122
*MTWAI–maximal value throughout follow-up*				
Median, q25**–**q75, Min**–**max	4, 2–8.5, 0–19	7.5, 3–9, 0–13	5, 2–9, 0–17	0.704
Reported flare* No (n = 284) Yes (n = 406)	213 (41.3%) 303 (58.7%)	7 (50.0%) 7 (50.0%)	64 (40.0%) 96 (60.0%)	0.753
*Flare possibly or highly related to (focus on yes):*				
NSAIDs Antibiotics GI tract infection Other infection Treatment decr. / disc Other medication	19 (6.3%) 6 (2.0%) 51 (16.8%) 29 (9.6%) 74 (24.4%) 9 (3.0%)	0 (0%) 1 (14.3%) 1 (14.3%) 0 (0%) 2 (28.6%) 0 (0%)	4 (4.2%) 1 (1.0%) 22 (22.9%) 10 (10.4%) 22 (22.9%) 5 (5.2%)	0.746 0.150 0.347 0.924 0.864 0.484
*Flare management (focus on the yes):*				
Hospitalization Ambulatory Surgery Drug therapy	39 (12.9%) 89 (29.4%) 16 (5.3%) 271 (89.4%)	0 (0%) 1 (14.3%) 0 (0%) 7 (100%)	7 (7.3%) 14 (14.6%) 3 (3.1%) 90 (93.8%)	0.278 **0.008** 0.702 0.367
*Focus on the hospitalizations:*				
Total days of hosp. Median, q25**–**q75, Min**–**max	4, 0–16, 0–90	–	0, 0–3, 0–12	0.063

Bold indicates a significant *p*-value (*p* < 0.05)

**Table 7:** Demographic and clinical characteristics for the different genotypes of rs4353135 in UC patients

	rs4353135 TT	rs4353135 GT	rs4353135 GG	*p* value (Fisher or Kruskal–Wallis)
*Gender*				
Male (n = 369) Female (n = 321)	169 (53.3%) 148 (46.7%)	165 (55.2%) 134 (44.8%)	35 (47.5%) 39 (52.7%)	0.474
*Age at diagnosis [years]*				
Median, q25**–**q75, Min**–**max	27.4, 19.7–38.4, 3.2–79.6	28.7, 20.4–38.4, 2.9–78.1	29.3, 19.2–42.6, 3.9–72.1	0.628
*Disease duration [years]*				
Median, q25**–**q75, Min**–**max	10.9, 6.9–18.4, 0.3–49.5	11.5, 6.9–17.6, 0.2–48.7	13.1, 6.9–22.5, 1.0–45.3	0.580
Complications*				
No (n = 286) Yes (n = 404)	135 (42.6%) 182 (57.4%)	125 (41.8%) 174 (58.2%)	26 (35.1%) 48 (64.9%)	0.507
MTWAI**—maximal value throughout follow-up				
Median, q25**–**q75, Min**–**max	4, 2–9, 0–19	5, 2–9, 0–18	4, 2–7, 0–14	0.129
Reported flare*				
No (n = 284) Yes (n = 406)	121 (38.2%) 196 (61.8%)	130 (43.5%) 169 (56.5%)	33 (44.6%) 41 (55.4%)	0.329
*Flare possibly or highly related to (focus on yes):*				
NSAIDs Antibiotics GI tract infection Other infection Treatment decr./disc Other medication	11 (5.6%) 4 (2.0%) 32 (16.3%) 23 (11.7%) 50 (25.5%) 4 (2.0%)	11 (6.5%) 4 (2.4%) 34 (20.1%) 11 (6.5%) 41 (24.3%) 9 (5.3%)	1 (2.4%) 0 (0%) 8 (19.5%) 5 (12.2%) 7 (17.1%) 1 (2.4%)	0.705 1.000 0.632 0.168 0.543 0.219
*Flare management (focus on the yes):*				
Hospitalization Ambulatory Surgery Drug therapy	24 (12.2%) 67 (34.2%) 8 (4.1%) 178 (90.8%)	20 (11.8%) 30 (17.8%) 10 (5.9%) 150 (88.8%)	2 (4.9%) 7 (17.1%) 1 (2.4%) 40 (97.6%)	0.443 **0.001** 0.692 0.248
*Focus on the hospitalizations:*				
Total days of hosp. Median, q25**–**q75, Min**–**max	6.5, 2.5–18.5, 0–90	0, 0–7, 0–41	3, 0–6, 0–6	**0.038**

Bold indicates a significant *p*-value (*p* < 0.05)

**Table 8:** Demographic and clinical characteristics for the different genotypes of rs55646866 in UC patients

	rs55646866 CC	rs55646866 CT	rs55646866 TT	*p* value (Fisher or Kruskal–Wallis)
*Gender*				
Male (n = 369) Female (n = 321)	302 (53.3%) 265 (46.7%)	63 (56.8%) 48 (43.2%)	4 (33.3%) 8 (66.7%)	0.302
*Age at diagnosis [years]*				
Median, q25**–**q75, Min**–**max	28.0, 19.9–38.7, 2.9–79.6	30.7, 22.1–38.8, 3.9–69.4	18.3, 8.5–22.1, 3.4–49.2	**0.004**
*Disease duration [years]*				
Median, q25**–**q75, Min**–**max	11.1, 6.7–18.1, 0.2–49.5	13.2, 7.8–20.0, 0.5–38.3	10.2, 7.0–14.2, 4.6–40.1	0.192
Complications*				
No (n = 286) Yes (n = 404)	244 (43.0%) 323 (57.0%)	39 (35.1%) 72 (64.9%)	3 (25.0%) 9 (75.0%)	0.160
*MTWAI—maximal value throughout follow-up*				
Median, q25**–**q75, Min**–**max	5, 2–9, 0–19	5, 2–9, 0–17	1.5, 0–2.5, 0–14	**0.009**
Reported flare*				
No (n = 284) Yes (n = 406)	230 (40.6%) 337 (59.4%)	49 (44.1%) 62 (55.9%)	5 (41.7%) 7 (58.3%)	0.777
*Flare possibly or highly related to (focus on yes):*				
NSAIDs Antibiotics GI tract infection Other infection Treatment decr./disc Other medication	20 (5.9%) 6 (1.8%) 57 (16.9%) 32 (9.5%) 86 (25.5%) 10 (3.0%)	3 (4.8%) 2 (3.2%) 15 (24.2%) 5 (8.1%) 10 (16.1%) 4 (6.5%)	0 (0%) 0 (0%) 2 (28.6%) 2 (28.6%) 2 (28.6%) 0 (0%)	1.000 0.444 0.249 0.223 0.232 0.413
*Flare management (focus on the yes):*				
Hospitalization Ambulatory Surgery Drug therapy	42 (12.5%) 99 (29.4%) 17 (5.0%) 302 (89.6%)	4 (6.5%) 5 (8.1%) 2 (3.2%) 59 (95.2%)	0 (0%) 0 (0%) 0 (0%) 7 (100%)	0.375 ** < 0.001** 0.822 0.376
*Focus on the hospitalizations:*				
Total days of hosp. Median, q25**–**q75, Min–max	4, 0–14, 0–90	0, 0–6, 0–12	–	0.225

Bold indicates a significant *p*-value (*p* < 0.05)

## Discussion

Polymorphisms in the regulatory region of *NLRP3* have been associated with an increased risk to develop CD, yet the impact of these variants on established IBD has not been clarified, so far. We here analyzed for possible associations between the SNPs rs10733113, rs4353135 and rs55646866 and the clinical characteristics of patients from the SIBDCS. Homozygous genotype for the major allele for rs10733113 was associated with a lower CDAI in CD patients. The same trend could be observed for rs4353135 and rs55646866. The SNPs rs4353135, rs55646866 and rs10733113 are in close proximity in the downstream region of NLRP3. Possible linkage disequilibrium between SNP pairs has been reported previously [1]: while D’ values indicated linkage disequilibrium for all SNP pairs, r^2^ showed very low linkage for 2 of the 3 SNP pairs. However, our calculations of r^2^ values with SIBDCS data showed no indication of substantial linkage disequilibrium among the three studied SNPs. Furthermore, linear regression models showed log-additive allele effects on maximal CDAI, suggesting independent effects of SNPs’ alleles Similarly, additive effects and a better diagnostic value of a genetic risk score in comparison to individual SNPs have recently been reported by Cleynen et al. [18]. As the CDAI describes the disease activity in CD patients, our findings suggest that the investigated CD-associated *NLRP3* variants are associated with a less severe course of disease. In addition, homozygous genotype for the major allele for rs10733113 was associated with fewer operations due to stenosis supporting our finding of an overall less severe course of disease in patients of this genotype.

The investigated polymorphisms in the regulatory region of *NLRP3* had been associated to CD, only, but not to UC [1]. Still, as similarly elevated IL-1β levels have been reported for UC patients, suggesting that alterations in NLRP3 inflammasome activity and IL-1β levels might also affect clinical disease characteristics in UC patients, UC patients were also included in our analysis. In contrast to CD, UC patients homozygous for the major allele of the studied *NLRP3* polymorphisms seem to have a more severe course of disease. Homozygous genotype for all three polymorphisms was significantly associated with more days of hospitalization indicating most likely an acute flare. Again, the association with all three SNPs could not be explained by linkage disequilibrium, as we observed an additive risk for the genetic risk score for the number of ambulatory visits and did not find r^2^ values indicative for linkage disequilibrium of these SNPs in the SIBDCS. In addition, homozygous genotype (GG) for the major allele of rs55646866 was associated with a higher MTWAI. As the MTWAI characterizes disease severity in UC patients, this finding further suggests a more severe course of disease in UC patients with GG genotype. In contrast, this genotype was associated with a higher age at diagnosis of disease. Higher age at diagnosis might suggest that the GG variant of rs55646866 is protective against development of UC, but we observed no further results that would support such a finding.

Further clinical characteristics, e.g. other parameters indicative of more severe disease like type of medication or need of treatment intensification, effects on CRP or calprotectin levels or nutrient deficiencies, were not affected by *NLRP3* variants, neither in CD nor in UC patients.

Genetic determination of high interindividual variation of in vitro IL-1β secretion and association with disease course in CD and UC patients has been previously reported [19, 20]. So far, little is known about the functional consequences of the investigated SNPs in the regulatory region of *NLRP3*. *NLRP3* mRNA expression was lower in PBMCs isolated from carriers of the major alleles of rs4353135 and rs10733113 in the CD-cohorts studied by Villani et al. and in patients with myocardial infarction and healthy controls [1, 13]. IL-1β secretion from isolated PBMCs was lower in patients with homozygous genotype for the major allele (GG) of SNP rs6672995 [1] and the same trend could be observed for homozygous genotype for the major alleles of rs55646866 and rs10733113. Homozygous genotype for the minor allele of rs4353135 was further associated with a higher risk for oligoarticular and polyarticular juvenile idiopathic arthritis and higher levels of inflammatory markers in a Taiwanese population [21], while presence of the G-allele of rs10733113 was associated with early onset disease in psoriatic arthritis [22].

These findings suggest that the *NLRP3* polymorphisms that are associated with CD might lead to lower NLRP3-inflammasome activity and lower IL-1β secretion. In line with the proposed pro-inflammatory role for NLRP3 and IL-1β, such lower NLRP3 activity and IL-1β secretion, might indeed be causally related to a less severe disease course resulting in a lower CDAI in CD patients homozygous for the major allele for these SNPs. As we observed a cumulative effect of the investigated *NLRP3* variants on CDAI it would be of particular interest to investigate whether being homozygous for the major allele for more than one of the investigated SNPs has a cumulative effect on IL-1β secretion, thus that patients homozygous for all three major alleles would have the lowest IL-1β levels. Furthermore, polymorphisms in the *IL1B* gene have been reported to affect course and severity of IBD [23] and nucleotide-binding domain and leucine-rich repeat caspase recruitment domain 4 (NLRC4) and NLRP3 can be recruited to the same macromolecular inflammasome complex [24]. Although no associations between *NLCR4* or *CASP1* polymorphisms and IBD have been reported so far, it would be very interesting to investigate whether IL-1β levels are affected by cumulative effects of polymorphisms in *IL1B* itself and further genes involved in IL-1β processing. Unfortunately, such an analysis is beyond the scope of our study.

CD and UC are distinct clinical entities with differences in disease location, histology and only partly shared genetic susceptibility [25]. Still, the diverging effect of CD-associated *NLRP3* polymorphisms on disease characteristics in CD patients and UC patients is striking, as similarly elevated secretion levels of IL-1β have been observed in CD and UC patients [4–6, 8, 26]. The diverging role of *NLRP3* polymorphisms on clinical parameters in CD and UC patients remains unexplained and the role of NLRP3 and IL-1β in IBD is still elusive, as reviewed in detail recently [27]. Although consistently elevated levels of IL-1β secretion have been reported for both CD and UC patients, IL-1β does not seem to play a decisive role in the inflammatory process of IBD patients, as only patients with IL-10R deficiency responded to IL-1R-antagonist treatment [27]. Data from murine models of experimental colitis with animals lacking NLRP3 show diverging effects depending on the model, experimental conditions and environment. Both, aggravated as well as less severe course of disease have been reported for *Nlrp3*^*−/−*^ mice during acute DSS and TNBS colitis [9, 10, 12, 28, 29]. Itani et al. describe a more severe course of disease of *Nlrp3*^*−/−*^ mice during oxazolone colitis [11], while Mak’Anyengo et al. observed reduced colitis in *Nlrp3*^*−/−*^ with the T-cell transfer colitis model [30]. These findings may be explained by the different roles of IL-β during intestinal inflammation: Mak’Anyengo et al. showed NLRP3 dependent IL-1β promoted Th17 differentiation and GM-CSF production of T-cells acting pro-inflammatory and resulting in aggravated colitis. Bersudsky et al. on the other hand, demonstrated that not IL-1β but IL-1α is the decisive pro-inflammatory mediator in the DSS colitis model, whereas IL-1β supports epithelial cell proliferation and restoration of colon barrier [31].

Data from patients or animals with NLRP3 hyper-activity provide a similar controversial picture for the role of NLRP3 in colitis. Both aggravation as well as amelioration of colitis have been shown in patients and mice with various reasons of NLRP3 hyperactivation. Lack of negative regulation by IL-10 leads to NLRP3 overactivation in *Il10*^−/−^ mice that develop spontaneous colitis [32, 33]. In this model the pro-inflammatory role of IL-1β seems to be non-redundant as treatment with a CASP1 or a NLRP3 inhibitor ameliorated established disease and prevented or delayed development of spontaneous colitis. Similarly, lack of negative regulation of NLRP3 by mutated CARD8 was found to be responsible for development of CD in three related patients, as disease was responsive to IL-1β inhibition [34].

On the contrary, situations with protective effects of NLRP3 hyperactivation have been observed in mice carrying an activating, autoimmune-associated mutation of *Ptpn22* [29]. As NLRP3 is activated by PTPN22-dephosphorylation, the *Ptpn*^*619W*^ variant leads to increased NLRP3 activation and IL-1β secretion. Mice carrying this mutation were protected from DSS colitis. Likewise, mice carrying the same activating mutation (*Nlrp3*^R258W^) that is responsible for increased NLRP3 activation and IL-1β levels in patients with cryoporin associated periodic syndrome (CAPS) were resistant to DSS colitis and T cell transfer colitis [35]. In these animals NLRP3 hyperactivation led to remodelling of the gut microbiota and increased production of regulatory T cells and antimicrobial peptides.

As gut microbiota alterations have been reported for animal models of both lack [10] or hyperactivation [35] of NLRP3, the question arises, whether NLRP3 variant induced modulation of the gut microbiota might be involved in the effect on IBD disease course. Yet, Yilmaz et al. [36] performed an extensive analysis of microbiota disturbances in the SIBDCS, but did not report effects of polymorphisms and we are not aware of any other study investigating the impact of NLRP3 SNPs on the gut microbiota.

So far, it is not clear whether polymorphisms that affect IBD risk also contribute to disease course and severity. While O’Donnell et al. [37] report association of 8 SNPs with time to-abdominal surgery, Lee et al. [38] could not identify any associations of disease susceptibility loci with disease prognosis, but identified four loci with significant association to the course of CD without any association to disease susceptibility. In addition, TLR SNPs that do not affect disease susceptibility, were found to be associated with extensive colonic disease in both CD and UC patients [39], and polymorphisms associated with smoking behavior could predict the number of surgeries and the time to first intestinal surgery in smoking Crohn’s disease patients [40].

Studies addressing the association of genetic loci to disease prognosis included a smaller number of patients in contrast to large genome wide association studies addressing IBD risk. We here studied 981 CD and 690 UC patients leading to significant associations, not robust to Bonferroni correction. Therefore, the identified associations between *NLRP3* polymorphisms and IBD prognosis need to be confirmed in additional cohorts.

Based on the currently available (sparse) data, that homozygous genotype for the major allele of CD associated *NLRP3* variants leads to lower *NLRP3* mRNA levels and activity resulting in lower IL-1β secretion, our findings suggest that during established CD the pro-inflammatory role of IL-1β might dominate, thus that patients homozygous for the major allele of these SNPs might profit from a less severe course of disease. In UC patients on the other hand, our findings support a protective role of NLRP3 such that homozygous genotype for the major alleles of the investigated *NLRP3* polymorphisms might favour a more severe course of disease.

The fact, that the major alleles of *NLRP3* polymorphisms are the CD associated alleles implies that a considerable number of CD patients—in our study 43%—might profit from the beneficial effect on disease severity. On the other hand, in CD patients homozygous for the minor allele, potentially resulting in a more active NLRP3 inflammasome, newly available NLRP3 inhibitors, e.g. MCC950 or Cy-09 [41, 42] might present a novel treatment option. In contrast, based on our findings that the major alleles of CD-associated *NLRP3* variants were associated with more days of hospitalization in UC patients, treatment with NLRP3 inhibitors might be detrimental in UC patients. Nevertheless, a detailed analysis of the effects of *NLRP3* variants on NLRP3-inflammasome activity and on IL-1β cytokine levels in IBD patients is necessary, before considering *NLRP3* variant data for treatment decisions.

## Conclusions

In summary, we here show that SNPs in the regulatory region of the *NLRP3* gene have diverging effects on the course of disease in CD and UC patients. Our findings should be taken into account, if treatment with novel NLRP3 inhibitors is considered. Yet, further confirmation of the impact of *NLRP3* polymorphisms on IBD disease course in additional and/or larger cohorts and a more detailed analysis of the consequences of *NLRP3* variants is necessary.

## Supplementary Information

**Additional file 1.** Figure S1.

**Additional file 2.** Table S1.

## Data Availability

Data may be made available upon request due to ethical restrictions imposed by the Swiss IBD cohort study group which collected this data. Interested researchers may request access to the collected data from this study in the same manner as the authors did. Data is available upon ethical approval and a request to the Head of the cohort, Dr. Gerhard Rogler, at gerhard.rogler@usz.ch. Data access requests may also be made to the Swiss IBD Cohort at sibdcs-submission@chuv.ch or http://ibdcohort.ch/index.php/informationen-fuer-forscher.html.

## Abbreviations

NLRP3: NOD-like receptor pyrin domain containing 3
SNP: Single nucleotide polymorphism
SIBDCS: Swiss IBD cohort study
CD: Crohn’s disease
UC: Ulcerative colitis
PAMP: Pathogen associated molecular pattern
DAMP: Danger associated molecular pattern
NOD: Nucleotide binding oligomerization domain
LRR: Leucine rich repeat
NLR: NOD-like receptor
CDAI: Crohn’s disease activity index
MTWAI: Truelove and Witts activity index
NLRC4: Nucleotide-binding domain and leucine-rich repeat caspase recruitment domain 4
