# Supplementary material for: Genotype–phenotype associations of polymorphisms within the gene locus of NOD-like receptor pyrin domain containing 3 in Swiss inflammatory bowel disease patients
Source: BMC Gastroenterol. 2021 Aug 3;21:310. doi: 10.1186/s12876-021-01880-9 (PMC8336111; doi:10.1186/s12876-021-01880-9)
Supplement: Supplementary file 1 — Additional file 1. Figure S1. [file 12876_2021_1880_MOESM1_ESM.pptx]

## Slide 1
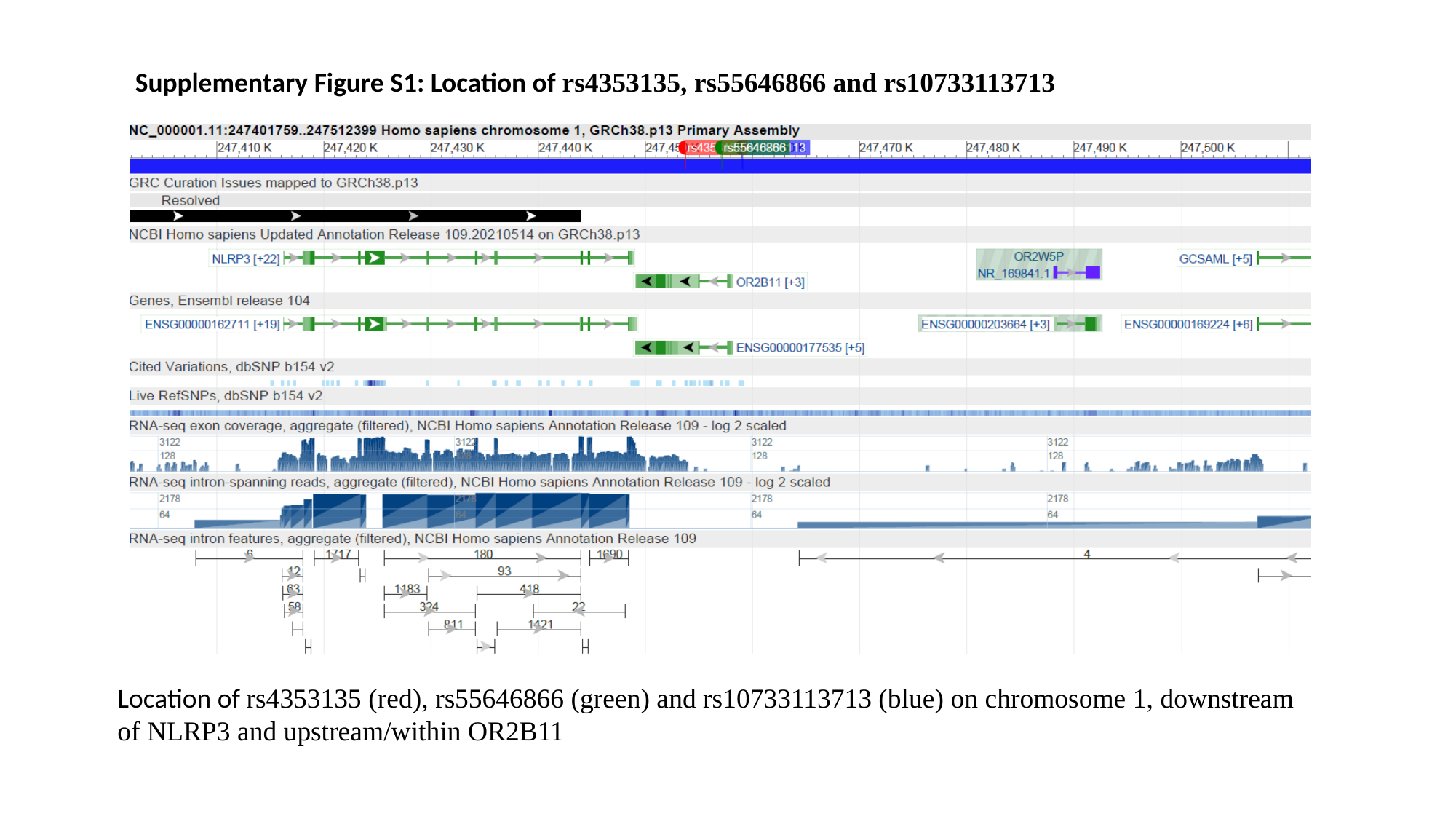

Supplementary Figure S1: Location of rs4353135, rs55646866 and rs10733113713
Location of rs4353135 (red), rs55646866 (green) and rs10733113713 (blue) on chromosome 1, downstream of NLRP3 and upstream/within OR2B11
