# Supplementary material for: Genotype–phenotype associations of polymorphisms within the gene locus of NOD-like receptor pyrin domain containing 3 in Swiss inflammatory bowel disease patients
Source: BMC Gastroenterol. 2021 Aug 3;21:310. doi: 10.1186/s12876-021-01880-9 (PMC8336111; doi:10.1186/s12876-021-01880-9)
Supplement: Supplementary file 2 — Additional file 2. Table S1. [file 12876_2021_1880_MOESM2_ESM.docx]

**Supplementary Tables:**

**Supplementary Table S1: Major Allele Frequencies in different populations**

|  | rs4353135 | | rs55646866 | | rs10733113 | |
| --- | --- | --- | --- | --- | --- | --- |
|  | IBD | General population | IBD | General population | IBD | General population |
| SIBDCS | 0.68 |  | 0.89 |  | 0.74 |  |
| European  (Villani *et al.*) | 0.71* | 0.64 | 0.96* | 0.85 | 0.90* | 0.83 |
| Canada  (Villani *et al.*) | 0.73* | 0.65 | 0.90* | 0.84 | 0.90* | 0.78 |
| American |  | 0.72 |  | 0.90 |  | 0.86 |
| European |  | 0.68 |  | 0.88 |  | 0.85 |
| African |  | 0.77 |  | 0.97 |  | 0.73 |
| Asian |  | 0.56 |  | 0.94 |  | 0.97 |
| South Asian |  | 0.71 |  | 0.97 |  | 0.92 |
| Latin American |  | 0.73 |  | 0.88 |  | 0.85 |

* = CD patients

### Compilated from Villani *et al.^1^*, the ALFA project (*L. Phan, Y. Jin, H. Zhang, W. Qiang, E. Shekhtman, D. Shao, D. Revoe, R. Villamarin, E. Ivanchenko, M. Kimura, Z. Y. Wang, L. Hao, N. Sharopova, M. Bihan, A. Sturcke, M. Lee, N. Popova, W. Wu, C. Bastiani, M. Ward, J. B. Holmes, V. Lyoshin, K. Kaur, E. Moyer, M. Feolo, and B. L. Kattman. "ALFA: Allele Frequency Aggregator." National Center for Biotechnology Information, U.S. National Library of Medicine, 10 Mar. 2020,* [www.ncbi.nlm.nih.gov/snp/docs/gsr/alfa/](http://www.ncbi.nlm.nih.gov/snp/docs/gsr/alfa/)*),* 1000 Genomes Project phase3 release V3+, and gnomAD

**Supplementary table 2:**


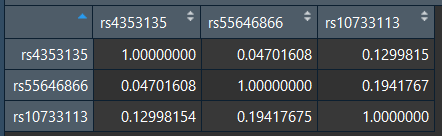


Pairwise linkage disequilibrium (measured by the square of the correlation coefficient (r2) sof investigated SNPs.

References:

1. Villani AC, Lemire M, Fortin G, et al. Common variants in the NLRP3 region contribute to Crohn's disease susceptibility. Nat Genet 2009;41:71-6.
